# University of Edinburgh. Prizes to Be Given by the Medical Faculty

**Published:** 1836-10

**Authors:** Thos. Stewart Traill

**Affiliations:** Dean of the Medical Faculty


UNIVERSITY OF EDINBURGH. PRIZES TO BE GIVEN BY THE MEDICAL FACULTY.

I. The Medical Faculty resolve, That they will present annually three gold medals to the medical graduates of the university, for the best thesis on three specified subjects.

II. That the first presentation shall take place on the graduation-day of 1837.

III. That the following shall be the subjects of competition for that year:

1. An Experimental Inquiry into the Physiology of Cutaneous Absorption, with its application to Therapeutics.

2. An account of the cases, or of any class of cases, occurring in the medical clinical wards attached to the university, during any quarterly period between 1st November, 1835, and 1st May, 1837, with the exception of the autumn of 1836.

3. An account of the cases, or of any class of cases, occurring in the surgical clinical wards attached to the university, during any quarterly period between 1st November, 1835, and 1st May, 1837, with the exception of the autumn of 1836.

IV. That the competing Theses shall be transmitted to the Dean of the Medical Faculty on or before 1st June, 1837.

V. That each Thesis shall be accompanied with a sealed packet, inclosing the following declaration, signed by the Author:

"I hereby declare on my honour, that in composing the annexed treatise, I have received no aid from any individual."

To which in the case of experiments he will add,—"and that the experiments described in it were performed with my own hands, and without any other assistance except what was indispensable for their performance."

The Thesis and sealed packet to be identified by a motto, and written in a fair and legible hand, and in such manner as that the author cannot be known, till the sealed packet be opened. The language to be English, Latin, or French.

VI. That in the case of experiments the author of the Thesis pronounced to be the best may be called on to repeat and authenticate his results before a Committee of the Faculty.

VII. That the Faculty reserve to themselves the power of withholding any of the medals, if it shall appear to them that none of the Theses on one or more of the specified subjects is worthy of being so distinguished.

VIII. That the Competitors shall transmit to the Dean, at the same time with the other Candidates, the usual Thesis on any subject different from those specified for the Competition.

IX. That the Faculty will further bestow a fourth Gold Medal on the author of any Thesis on a subject chosen by himself, which may appear to them the best of the Thesis of that class, and worthy of receiving the honour.

X. That the successful Thesis shall in every case be published by their respective authors, with an appropriate title designating them as the Prize Dissertations for the year.

Thos. Stewart Traill,
*Dean of the Medical Faculty.*

